# Acrofacial necrotic ulcers in an infant: An undiagnosed presentation

**DOI:** 10.3389/fped.2022.1069242

**Published:** 2022-12-22

**Authors:** Georgina-Maria Sarika, Rony Shreberk-Hassidim, Alexander Maly, Vered Molho-Pessach

**Affiliations:** ^1^ Department of Dermatology; ^2^ Department of Pathology, Hadassah Medical Center, The Faculty of Medicine, Hebrew University of Jerusalem, Jerusalem, Israel

**Keywords:** acrofacial necrotic ulcers, perniosis, lipodystrophy, type 1 interferonopathies, undiagnosed cases

## Abstract

Acral necrotic ulcers in infancy are rare but have been described in type I interferonopathies. Herein, we present a case of an 8-year-old child who presented at the age of one month with severe ulceronecrotic lesions on the face and limbs with exacerbations following exposure to cold weather. Despite extensive investigation the case remains undiagnosed to this day. We hypothesize that this case represents a novel and yet unknown autoinflammatory disease.

## Introduction

Cold induced pernio-like lesions with necrotic ulcers are rare in infancy and are usually associated with an underlying genetic autoinflammatory disease. Aicardi-Goutières syndrome (AGS), STING-associated vasculopathy with onset in infancy (SAVI), PLCG2-associated antibody deficiency and immune dysregulation (PLAID) and Proteasome-associated autoinflammatory syndromes (PRAAS); such as Nakajo-Nishimura syndrome (NNS) and Chronic Atypical Neutrophilic Dermatosis with Lipodystrophy (CANDLE), are some of the recognized cold-associated syndromes, that are characterized by acral ulceronecrotic lesions (Table 1). These autoinflammatory disorders are referred to as type I interferonopathies and are due to inborn errors in interferon (IFN) signaling, resulting in upregulation of IFN*α* (1–7). Patients suffering from these disorders tend to present early in life. Despite the distinct clinical phenotypes of each disorder in this group, clinical manifestations may overlap and are associated with high morbidity and mortality without treatment (8). Other conditions that might present with similar manifestations include prolidase deficiency, lupus erythematosus, systemic sclerosis, chilblains (pernio), cryoglobulinemia, cryofibrinogenemia and pyoderma gangranosum (9, 10).

**Table 1:** Characteristics of type I interferonopathies included in our differential diagnosis.

Syndrome	**AGS^1,2^**	**SAVI^3^**	**PLAID^4^**	**NNS^5,7^**	**CANDLE^6,7^**
**Affected gene**	*TREX1* *RNASEH2A* *RNASEHSB* *RNASEH2C* *SAMHD1* *ADAR1* *IFIH1*	*STING1*	*PLCG2*	*PSMB8* (Belongs to the Proteasome-associated autoinflammatory Syndromes)	*PSMB8* (Belongs to the Proteasome-associated autoinflammatory Syndromes)
**Inheritance**	Autosomal recessive	Autosomal dominant	Autosomal recessive	Autosomal recessive	Autosomal recessive
**Age of onset**	Most commonly: First weeks- 4 months of life Less commonly: Between 4 and 12 months of life	Infancy Most commonly first 6 months	Infancy	Early infancy	Early infancy or early childhood
**Dermatological manifestations**	Recurrent, cold induced necrotic pernio-like lesions on hands, feet, and ears	Acral violaceous nodules and plaques (fingers, toes, nose, ears) Limb ulcers Skin necrosis Atrophic plaques	Cold urticaria, Ulcerating granulomas, Spontaneous ulcerating nose lesions Recurrent small papules and erosions on fingers, toes, head +/ trunk	Cold induced pernio-like lesions in hands and feet Nodular erythema Heliotrope-like periorbital rash	Cold induced pernio like lesions Annular erythematous plaques on torso, face and limbs Erythematous swollen eyelids Chronic neutrophilic dermatosis
**Systemic manifestations**	Recurrent fevers Encephalopathy Developmental delay Motor disorders (dystonia, spasticity) Feeding difficulties Epileptic seizures Glaucoma Autoimmunity (thyroiditis) Join contractures Microcephaly Thrombocytopenia Intracranial calcifications	Interstitial lung disease Pulmonary fibrosis Hyperglobulinemia Increased CRP, ESR Positive ANA	Autoimmunity (thyroiditis, vitiligo) Positive ANA Recurrent URTI'S	Recurrent fevers Progressive lipodystrophy Calcification of the basal ganglia Hepatospleno-megaly Clubbed fingers and toes Arthritis Increased CRP, ESR Joint contractures Failure to grow Hyperglobulinemia	Recurrent fevers Progressive lipodystrophy Hepatosplenomegaly Increased liver enzymes Arthritis Anemia Increased CRP, ESR Failure to grow Joint contractures

AGS, aicardi-goutières syndrome; CANDLE, chronic atypical neutrophilic dermatosis with lipodystrophy; NNS, Nakajo-Nishimura syndrome; PLAID, PLCG2-associated antibody deficiency and immune dysregulation; SAVI, STING-associated vasculopathy with onset in infancy.

We report on an eight-year-old boy who has suffered since early infancy from cold-aggravated necrotic ulcers on the face and extremities. Despite extensive work-up, none of the aforementioned disorders have been confirmed and the case still remains undiagnosed.

## Case presentation

The patient is an eight-year-old male born to non-consanguineous parents of Arab origin. He has three healthy siblings, and his pregnancy and delivery were normal. In January 2014, at the age of 5 months he presented to the Emergency Department of our hospital with acrofacial ulceronecrotic plaques which appeared initially at the age of 1 month over the cheeks and nose with subsequent development of similar lesions over the hands and feet. The parents reported exacerbations upon exposure to cold temperatures. At his arrival, he was hemodynamically stable and alert with no fever.

**Figure 1:**
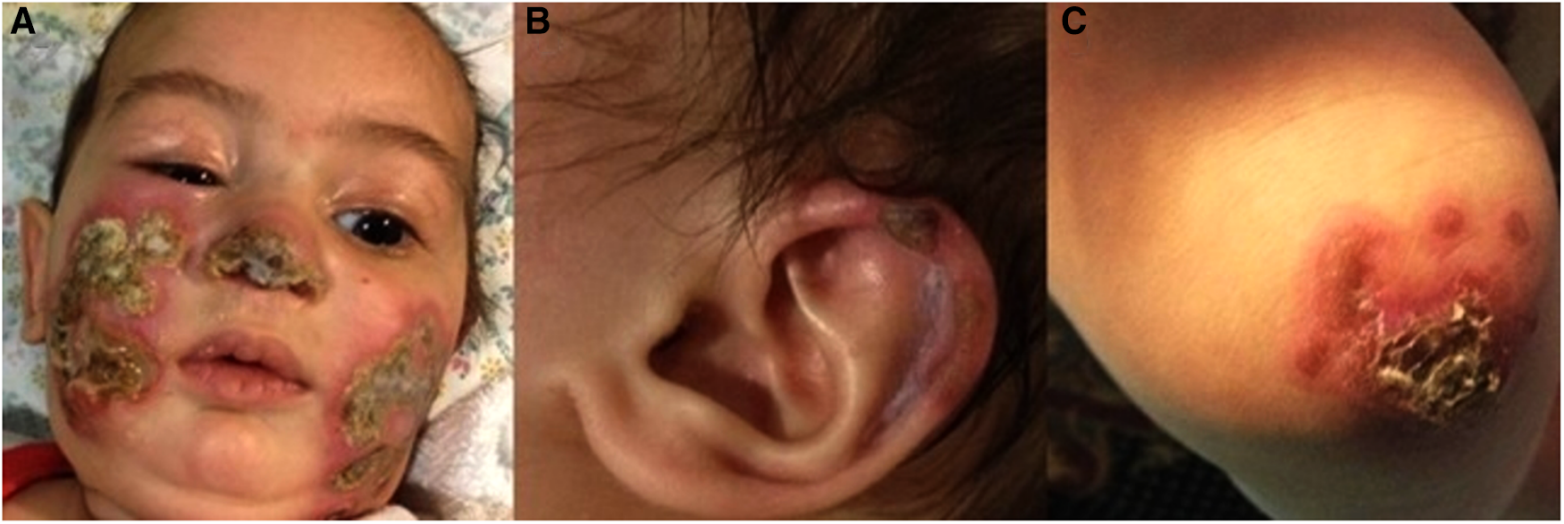
(**A–C**) Clinical features on admission at age 5-months: erythematous plaques with central necrosis on the tip of the nose and the cheeks bilaterally (**A**). Ulceration on the helix of the left ear (**B**) Right knee with erythematous plaques with yellow-brown central crusts (**C**).

Physical examination on admission showed erythematous violaceous indurated plaques with central necrotic ulceration located over the tip of the nose, bilateral cheeks and ear helices. Similar lesions were seen on the knees and extensor surfaces of the arms (Figures 1A–C). There were no cardiopulmonary, gastrointestinal, genitourinary or ophthalmological abnormalities.

The patient was admitted for further investigation and treatment. During his hospitalization he showed signs of poor feeding, weight loss and failure to thrive. A nasogastric tube was inserted that facilitated his feeding, improving his weight. Despite topical wound care and systemic antibiotics including cephalosporins and vancomycin, systemic corticosteroids and antifungals, no improvement was observed, with new lesions appearing on the right hand and right elbow after exposure to cold. In addition, the patient developed unexplained febrile episodes with concurrent deterioration of the ulcers. Extensive laboratory workup was performed, as follows: skin and blood cultures showed no evidence of microbial growth (bacteria, mycobacteria and fungi). Cutaneous leishmaniasis was also excluded by smear and polymerase chain reaction. Blood work showed normal kidney and liver function tests moderate leukocytosis and thrombocytosis, normal hemoglobin level and no marked elevation of C-reactive protein or erythrocyte sedimentation rate. Anti-nuclear antibodies, rheumatoid factor, anti-Ro, anti-La, immunoglobulins, cryoglobulins and cryofibrinogens, C3, C4, C-ANCA and *P*-ANCA were all within normal limit. A biochemical assay for prolidase deficiency was negative, chest x-ray, abdominal ultrasound, were normal. Serology for human immunodeficiency virus was negative as well.

**Figure 2:**
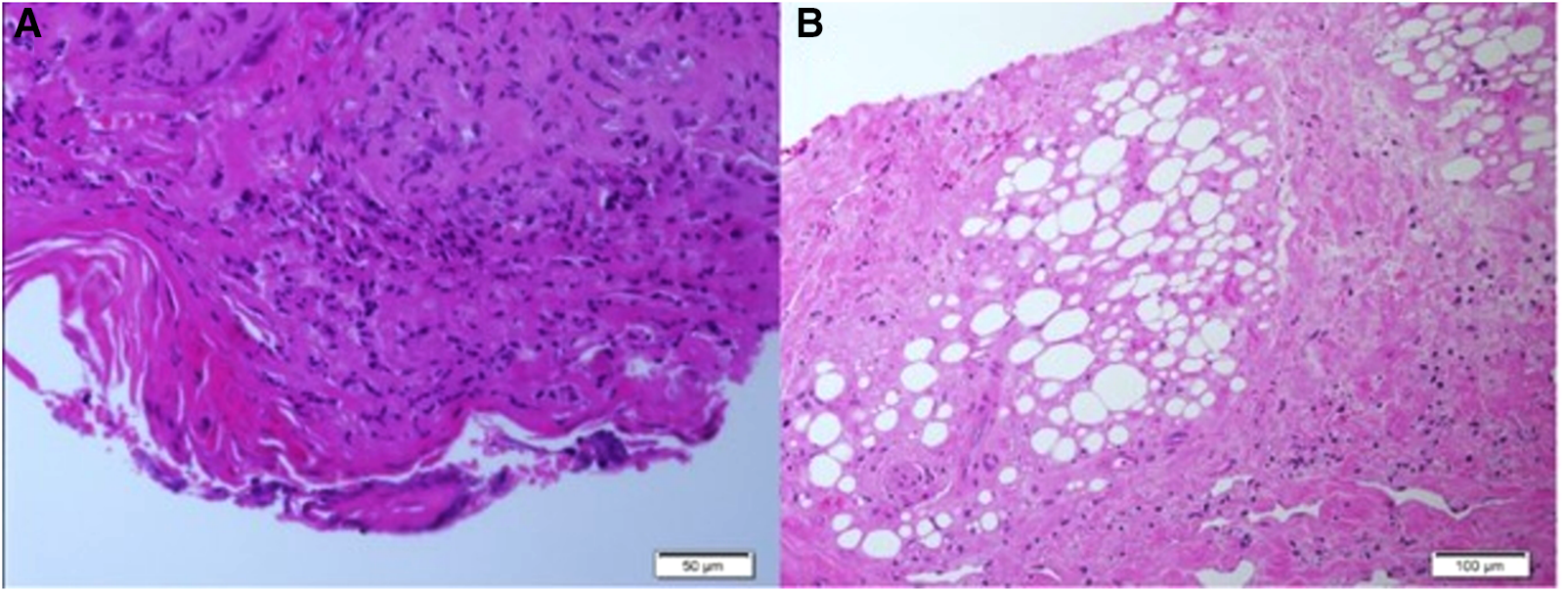
(**A,B**) Histopathological findings of skin biopsies: epidermal necrosis, a mononuclear perivascular and periadenxal infiltrate, fat necrosis with hyalinization. Changes compatible with lipodystrophy in the dermis and subcutaneous tissue. (Figure A: H&E, ×50, Figure B: H&E, ×100).

Two skin biopsies were performed, demonstrating epidermal necrosis, mild parakeratosis, mild focal vacuolar degeneration and apoptosis of basal cell keratinocytes., mononuclear lymphohistiocytic infiltrate surrounding blood vessels, eccrine glands and hair follicles together with fat necrosis and hyalinization suggestive of lipodystrophy. No thickening of the basement membrane was observed (Figures 2A,B).

Given the history of transient sterile pyrexias with pernio-like skin lesions, ulcerations and lipodystrophy, a disorder of type I interferonopathy was suspected. Trio exome sequencing obtained from peripheral blood of the patient and his parents was negative for mutations in genes responsible for different type-1 interferonopathies that were included in our differential diagnosis. Mutations in candidate genes which may explain the phenotype of our patient were not identified as well.

**Figure 3:**
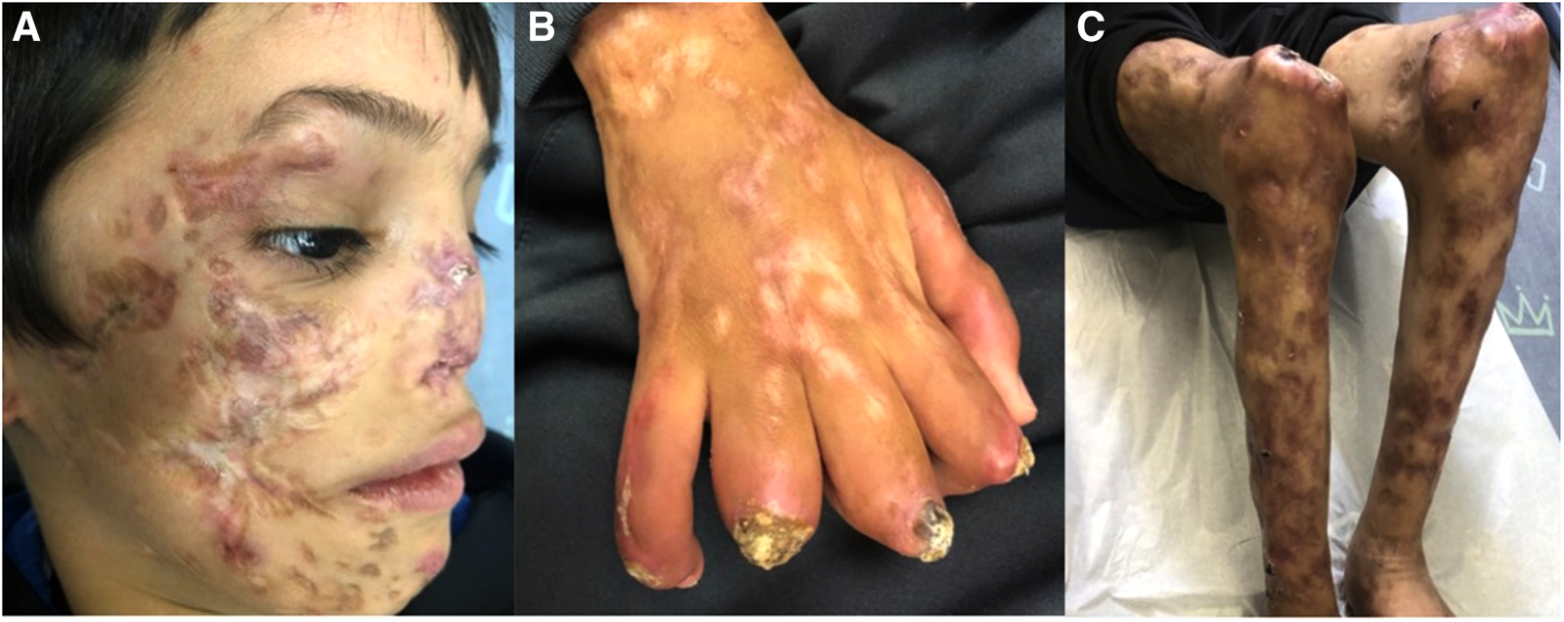
(**A–C**) Clinical features on his last follow up at the age of 9-years. Dyspigmented atrophic plaques on the cheeks, nose, chin and lateral upper eyelidswith deformity and scarring. Dystrophic and wasted saddle nose (**A**). Right hand showing ulcerated plaques covered with thick crust and scale, dyspigmented atrophic scars and contractures of fingers (**B**). Muscle wasting and dyspigmented atrophic scars on the lower limbs, ulcerated plaques and swelling of the knees (**C**).

Following prolonged admission and inconclusive investigations, the patient's diagnosis remained unclear. Severe scarring and deformity became noticeable with time. The patient was eventually transferred to a hospital in his hometown to continue follow up and rehabilitation. He returned for follow-up for the first time after his discharge in November 2021, 7 years later at the age of 8-years-old. Then, physical examination showed numerous dyspigmented atrophic scars on the face and ulcers covered with crusts and thick yellow scales on the extremities. Saddle nose, muscle wasting, lipodystrophy and joint contractures were noted. Sadly, on his recent follow up in our clinic in November 2022 he presented a similar but more strikingly debilitating picture (Figures 3A–C). There was no involvement of the trunk or the genital area. During winter months the patient continues to suffer from febrile episodes with worsening of his skin lesions.

## Discussion

We present here a rare and severe case of perniosis associated with lipodystrophy appearing in early infancy and associated with failure to thrive and arthropathy. Thorough laboratory and genetic investigations failed to reveal the cause for his severely disabling condition, although a mutation in a yet unknown gene is most likely. Unfortunately, insurance-related issues prevented us from performing genome sequencing to conclude his genetic workup.

Our primary differential diagnoses included the spectrum of type I interferonopathies, characterized by dysregulation of IFN*α* signaling leading to constitutive upregulation or downregulation of negative regulatory mechanisms of this pathway. These disorders tend to manifest with skin vasculopathy, chilblains and panniculitis/lipodystrophy, central nervous system disorders and interstitial lung disease. These features represent the “clinical IFN signature” (11); at least partially, our patient seems to share this clinical signature. Various methods were proposed in 2019 by Lamot et al., for type 1 interferon detection, however, none of these methods were available to perform in our clinical setting due to lack of medical insurance (12).

Type 1 interferonopathies are usually resistant to conventional treatments. However, two recent studies on the treatment of type-1 interferonopathies, including AGS and familial chilblain lupus, SAVI, NNS and CANDLE syndrome with Janus kinase (JAK) inhibitors, showed promising results with improvement of dermatological and systemic symptoms. There were no severe side effects reported, making it a safe treatment option in these difficult-to-treat and debilitating disorders (And while our patient's undiagnosed disease progresses into a chronic disability, we acknowledge the clinical similarities of his symptoms to those of the type I interferonopathies and consider if future empirical treatment with a JAK inhibitor is justified (13, 14).

To conclude, we presented a patient with early onset acral ulceronecrotic lesions associated with lipodystrophy. Unfortunately, despite our efforts, the patient currently remains undiagnosed and untreated. We hope that the publication of this unique case will enable others to identify similar cases and assist in solving this diagnostic and therapeutic challenge.

## Data Availability

The raw data supporting the conclusions of this article will be made available by the authors, without undue reservation.
